# Intraosseous contrast administration for emergency stroke CT

**DOI:** 10.1007/s00234-021-02642-w

**Published:** 2021-01-18

**Authors:** Hermann Krähling, Max Masthoff, Wolfram Schwindt, Christian Paul Stracke, Philipp Schindler

**Affiliations:** 1 Clinic for Radiology, University Hospital Muenster, Muenster, Germany; 2 Division of Interventional Neuroradiology, University Hospital Muenster, Muenster, Germany

**Keywords:** Intraosseous access, Intraosseous contrast administration, CT imaging, Acute stroke

## Abstract

Computed tomography (CT) imaging in acute stroke is an established and fairly widespread approach, but there is no data on applicability of intraosseous (IO) contrast administration in the case of failed intravenous (IV) cannula placement. Here, we present the first case of IO contrast administration for CT imaging in suspected acute stroke providing a dedicated CT examination protocol and analysis of achieved image quality as well as a review of available literature.

## Introduction

Emergency CT imaging in the setting of suspected stroke is an established approach to rule out cerebral infarction or cerebral hemorrhage in patients presenting with acute neurological symptoms [1, 2]. Rapid diagnosis of stroke conditions is critical as patients need early CT imaging after the onset of symptoms to enable targeted management (e.g., endovascular clot retrieval and/or intravenous thrombolysis) [1, 2]. Therefore, vascular access is of high importance for applying contrast agents enabling for dedicated stroke CT imaging, including CT angiogram (CTA) and CT perfusion (CTP). However, some patients do not permit rapid peripheral IV cannulation (e.g., centralization, obesity, previous therapies). In these cases of delayed or failed IV placement, IO access catheters offer an alternative access path [3].

Several professional guidelines recommend IO access as the first alternative for critically ill or injured patients in whom medication and/or volume administration is necessary to restore vital functions in the case of delayed or failed IV cannulation [3–6]. In recent years, the development of purely manual to (semi-)automatic drilling devices has resulted in increasingly familiar and safe handling [3, 4, 7]. The ideal puncture site for establishing the IO access must be selected depending on the patient’s age, the type of procedure, or puncture system used (manual, semi-automatic, automatic) and considering patient or other restrictions (osteosynthesis material, fracture, or vascular injury at the planned puncture site) [8, 9]. In general, the puncture site should meet the following requirements: a relatively thin cortical bone combined with a large medullary cavity, a surface that is as flat as possible, and simple anatomical landmarks for rapid and safe identification and puncture with as little risk of dislocation as possible [8]. Three insertion sites meet these criteria: the proximal tibia, the distal tibia, and the proximal humerus [8, 9]. Current guidelines generally recommend the proximal medial tibia as the first-choice puncture site when using the most common (semi-) automatic puncture systems and in all age groups [8, 9]. Regardless of the chosen puncture site, outflow occurs *via* venous sinusoids into the central veins in the bone marrow and from here via draining veins into the systemic circulation.

Technical placement of the IO access often takes place out of the hospital at the scene of the arriving emergency physician or on arrival at the emergency room while in our tertiary care center it is a task of the anesthetists. Briefly, after orientation to anatomical landmarks, the puncture site is disinfected and locally anesthetized. The drill device is then placed at a 90° angle, the cortical bone is pierced, and the needle is inserted into the medullary cavity according to the device’s manual. For position control, aspiration of bone barrow and injection of a test fluid bolus (e.g., 5–10 cc sodium chloride) should be performed. Finally, the IO access is securely fixed and the infusion line or contrast media injector can be connected similar to a standard peripheral venous access.

Despite increasing evidence of safe IO drug and volume administration, there is limited data regarding pressure contrast media injection (CMI) in emergency CT or potential CT protocol adjustments using an IO access catheter [4, 7, 10–12]. So far, there are no reports regarding IO CMI for emergency stroke CT imaging.

Here, we present the first case of IO contrast administration for CT in suspected acute stroke. Moreover, we provide a dedicated CT examination protocol and analysis of achieved image quality as well as a review of available literature.

## Case presentation

**Fig. 1:**
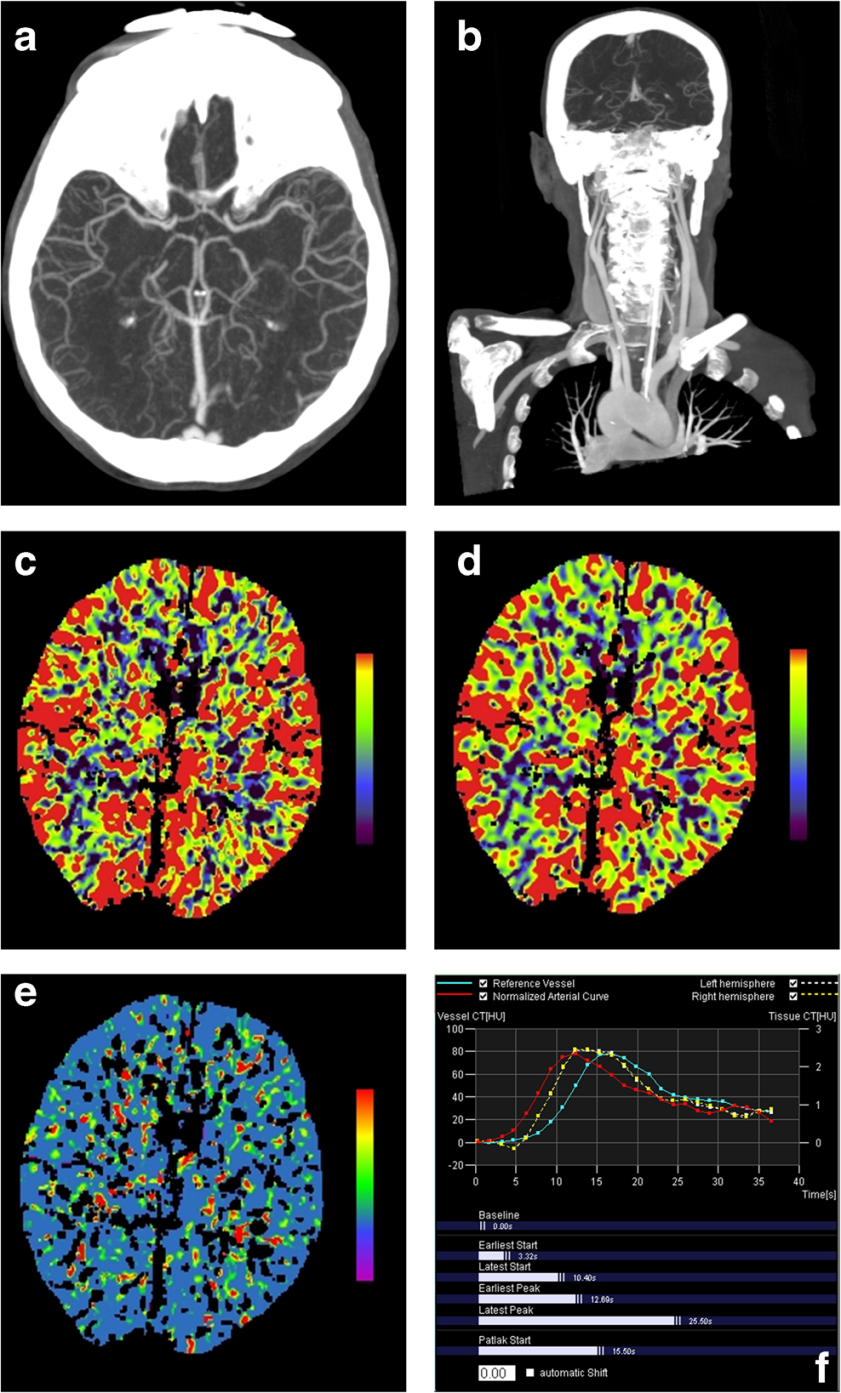
CTA and CTP via IO access. **a** Maximum intensity projection (MIP) axial reformatted image of the cerebral arteries. **b** Maximum intensity projection (MIP) coronal reformatted image of the supra-aortic branches. CTP post-processed maps of relative cerebral blood flow (CBF, **c**), cerebral blood volume (CBV, **d**), and mean transit time (MTT, **e**). (**f**) Technical adequacy confirmed by normal arterial input and venous outflow function curves with an adequate baseline and rapid upslope

A 54-year-old male patient presented to the emergency department with an unclear unconsciousness (Glasgow Coma Scale (GCS) 6). Technical placement of the tibial IO access took place out of the hospital at the scene of the arriving emergency physician due to failed IV cannulation. Laboratory parameters showed a low blood sugar level of 36 mg/dl indicating the presence of an acute hypoglycemia. The patient did not regain consciousness under repeated administration of glucose solution. During the administration of the glucose solution, a newly observed anisocoria appeared indicating the necessity of a pre-hospital intubation. After the arrival at our clinic and under persisting unconsciousness of the patient (GCS 6) assignment to stroke, CT was based on interdisciplinary consultation of an emergency physician, neurologist, and neuroradiologist to rule out cerebral infarction or cerebral hemorrhage. Contrast media was administered via the IO access while again IV cannulation failed. Stroke CT demonstrated the absence of an ischemic stroke, intracerebral bleeding, or another causal cerebral pathology (Fig. 1a–e). The patient regained consciousness after the CT scan and was later diagnosed with an insulin-dependent diabetes mellitus responsible for the acute hypoglycemia. The patient was released after a hospital stay of 15 days in a good general condition.

## Examination technique

**Fig 2:**
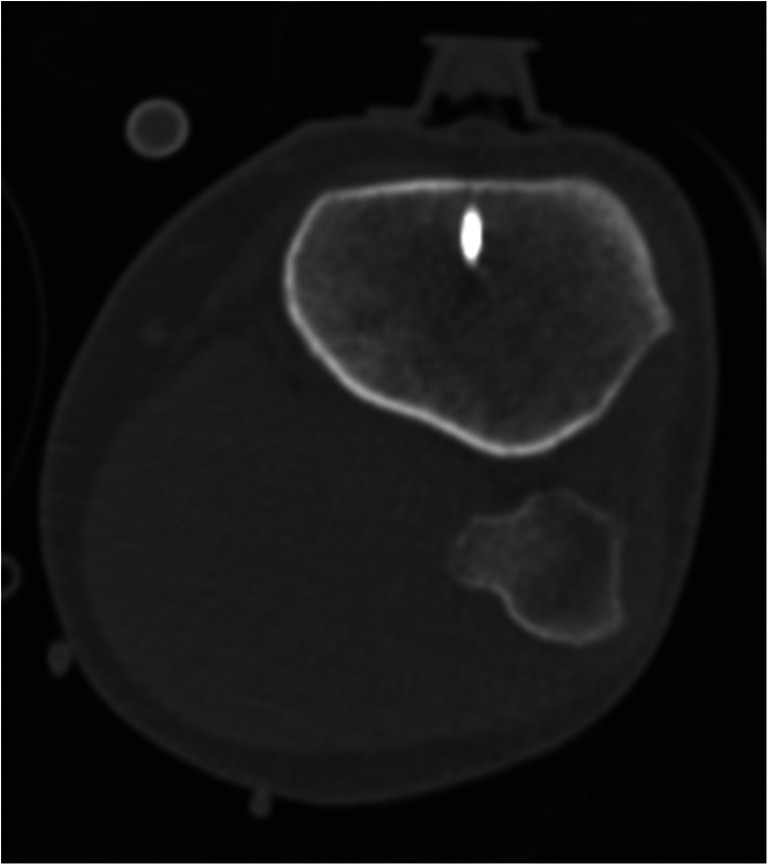
Short low-dose CT scan over the IO device. Exemplary, short CT scan over the IO device prior to the main examination to confirm correct placement within the intramedullary cavity (other patient)

Examination was performed with dual-source CT (SOMATOM Definition Flash, Siemens Healthineers, 128 × 0.6 mm) according to the identical IV institutional stroke CT protocol with the same amount and flow rate of iodinated non-ionic contrast medium (Ultravist, Bayer Healthcare) delivered by a power injector after non-enhanced CT of the brain. Our stroke protocol in case of an IO access additionally includes a short low-dose CT scan over the IO device prior to the main examination to confirm correct intramedullary placement (Fig. 2). The main examination starts with a non-contrast head CT scan followed by a CTA of the supra-aortic and cerebral vasculature (80 cc contrast medium, 4 cc/s flow rate) while the patients’ arms are lowered. CTP images were acquired after a delay of 180 s followed by an injection of 30 cc contrast medium at a flow rate of 5 cc/s. Acquisition parameters were 120 kV and 175 mA, rotation time 0.5 s, and pitch 0.5. Syngo VPCT Neuro Software (Siemens Healthineers) was used for analyzing raw perfusion data. The arterial input function and the venous output function were determined from the middle cerebral artery and the superior sagittal sinus. Maps of relative cerebral blood flow (CBF), cerebral blood volume (CBV), mean transit time (MTT), and time to drain (TTD) were calculated semi-automatically by software-based default settings as used in clinical routine.

## Image quality analysis

The IO CMI visually fulfilled the quality criteria of the institutional standard IV CMI stroke protocol rated by the performing technician and the physician. Technical adequacy of IO CT perfusion was confirmed by normal arterial input and venous outflow function curves with an adequate baseline and rapid upslope (Fig. 1f). CTA and CTP color maps were visually analyzed and rated by two senior neuroradiologists in a consensus reading by using a scoring system according to Abels et al. [13] The examination was considered to be of high quality and diagnostic value.

## Discussion

Here, we provide the first description of the feasibility of CMI via an IO access for CT in suspected acute stroke management. We demonstrated that IO CMI could be performed using established CT stroke protocols with identical contrast medium amount and flow rate comparable to IV CMI. In this case, no adverse events were observed, and a high image quality was achieved.

Cohen et al. firstly described the concept of CT with IO CMI using an animal model [7]. Two case reports by Ahrens et al. and Budach et al. demonstrated the feasibility of a CTA of the pulmonary arteries via IO access with 5 cc/s or CTA of the chest and abdomen with 4 cc/s, each with good quality and no adverse events [10, 12]. Winkler et al. also described the feasibility of thoracic CTA via IO cannula in a larger number of cases (*n* = 17) as a safe and effective route of CMI up to 4 cc/s [11]. Recently, the concept of CTA of the head-and-neck region via IO access was described in *n* = 4 cases in emergency trauma CT [4].

In the present case, we demonstrated the feasibility of IO contrast administration for CT in suspected acute stroke. Particularly, for the first time, a CTP of the head via IO CMI was performed. This underlines that the IO access is also suitable for complex neurologic CT examinations with high flow rates up to 5 cc/s via power injector.

This report focused on the feasibility of CT examination via IO access while the technical placement of the IO access was not the focus. As described elsewhere, we do recommend to perform a low-dose CT over the site of IO access as part of routine protocol prior to CMI to confirm correct placement within the intramedullary cavity while aspiration of bone marrow is not possible in all cases despite correct needle position (Fig. 2) [3, 4]. Antibiotics do not need to be administered routinely. The most common complication is extravasation of the applied contrast agent [14]. Severe complications such as fatty embolisms or osteomyelitis occur far less frequently and are rather associated to prolonged use of the IO access [14].

In case of failed IV cannulation, MRI or—in case of high suspicion of stroke—intra-arterial angiography without prior imaging are other valuable alternatives in emergency stroke management. Moreover, this technical note should be validated by further cases demonstrating cerebral infarction to analyze the quality of estimation of the hypoperfusion area as well as penumbra and ischemic core volume after IO CMI.

In conclusion, the current report demonstrates that IO access provides a fast and safe alternative for emergency CMI in stroke CT with good imaging quality. Furthermore, the first description of IO CMI in suspected acute stroke imaging using standardized routine protocols supports IO access as a suitable alternative for emergency cerebral vascular and perfusion imaging.
